# IcarisideII facilitates the differentiation of ADSCs to SCs via let-7i/STAT3 axis to preserve erectile function

**DOI:** 10.1186/s40659-019-0262-3

**Published:** 2019-10-03

**Authors:** Pingyu Ge, Yinxue Guo, Jun Shen

**Affiliations:** 1 Department of Urology Surgery, the First Affiliated Hospital of Guizhou University of Traditional Chinese Medicine, No. 71, Baoshan North Road, Guiyang, 550001 Guizhou China; 2 Department of Nephrology, the First Affiliated Hospital of Guizhou University of Traditional Chinese Medicine, Guiyang, Guizhou China

**Keywords:** Erectile function, ADSCs, let-7i, STAT3, ICAII

## Abstract

**Background:**

IcarisideII (ICAII) could promote the differentiation of adipose tissue-derived stem cells (ADSCs) to Schwann cells (SCs), leading to improvement of erectile function (EF) and providing a realistic therapeutic option for the treatment of erectile dysfunction (ED). However, the underlying molecular mechanisms of ADSCs and ICAII in this process remain largely unclear.

**Methods:**

ADSCs were treated with different concentrations of ICAII. Cell proliferation was determined by MTT assay. qRT-PCR and western blot were performed to detect expressions of SCs markers, signal transducer and activator of transcription-3 (STAT3), and microRNA-let-7i (let-7i). Luciferase reporter assay was conducted to verify the regulatory relationship between let-7i and STAT3. The detection of intracavernosal pressure (ICP) and the ratio of ICP/mean arterial pressure (MAP) were used to evaluate the EF in bilateral cavernous nerve injury (BCNI) rat models.

**Results:**

ICAII promoted cell proliferation of ADSCs in a dose-dependent manner. The mRNA and protein levels of SCs markers were increased by ICAII treatment in a dose-dependent manner in ADSCs. Moreover, let-7i was significantly decreased in ICAII-treated ADSCs and upregulation of let-7i attenuated ICAII-induced promotion of SCs markers. In addition, STAT3 was a direct target of let-7i and upregulated in ICAII-treated ADSCs. Interestingly, overexpression of STAT3 abated the let-7i-mediated inhibition effect on differentiation of ADSCs to SCs and rescued the ICAII-mediated promotion effect on it. Besides, combination treatment of ADSCs and ICAII preserved the EF of BCNI rat models, which was undermined by let-7i overexpression.

**Conclusion:**

ICAII was effective for preserving EF by promoting the differentiation of ADSCs to SCs via modulating let-7i/STAT3 pathway.

## Background

Prostate cancer is the second leading cause of cancer-related death in men worldwide [1, 2]. Erectile dysfunction (ED) administered by injury of cavernous nerve is a significant problem following prostate cancer surgery, which seriously affects the quality of life of patients [3–5]. Adipose tissue-derived stem cells (ADSCs) has significant treatment effect for ED in diabetics, post-prostatectomy patients, and those with peyronie’s disease [6, 7]. Icariside II (ICAII), isolated from traditional Chinese medicine Herba Epimedii, has been reported to improve erectile function (EF) in rats [8–11]. However, whether ICAII plays a protective role for EF via regulating the differentiation of ADSCs as well as the underlying molecular mechanisms have not been fully understood.

MicroRNAs (MiRNAs) have been reported to be associated with EF [12]. For example, it has been demonstrated that the expression levels of miR-93, miR-320 and miR-16 may be useful for the early diagnosis of ED in patients with diabetes [13]. miR-146a is related to the development of ED in patients with chronic prostatitis via targeting NOS1 [14]. In another report, miR-200a could participate in the mechanisms of aging-related via silent information regulator 1 inhibition [15]. These data suggested that determination of changes in miRNAs and related regulatory networks may open exciting avenues for improving the diagnosis and treatment options for ED. Although many efforts about the involvement of miRNAs in EF have been made, the investigations that miRNAs participate in the treatment of ICAII for ED remains largely unknown. Recently, microRNA-let-7i (let-7i) was reported to play vital roles in multiple diseases [16, 17]. Moreover, let-7i was also found be upregulated in bilateral cavernous nerve crush rats [18], whereas the functions of let-7i in ED remain largely unclear. Signal transducer and activator of transcription-3 (STAT3) has been widely reported to be associated with cancer progression, including ED [19, 20]. However, whether let-7i/STAT3 axis participates in the treatment of ICAII for ED by promoting the differentiation of ADSCs has not been revealed.

In this study, we sought to explore the roles of let-7i and STAT3 in the ICAII-mediated neuronal differentiation of ADSCs, as well as the effect of combination of ADSCs and ICAII on ED treatment.

## Materials and methods

### Isolation of ADSCs and differentiation of ADSCs to SCs

The experiments were approved by the Animal care and Experiments committee of the First Affiliated Hospital of Guizhou University of Traditional Chinese Medicine. The SD male rats (65–75 g, 4 weeks) were obtained from the Kaixue Biotechnology (Shanghai, China), following sacrificed. ADSCs were isolated from SD rats as descripted in previous study [2], subsequently cultured in Dulbecco’s Modified Eagle’s Medium/F12 (Gibco, Grand Island, NY, USA) supplemented with 10% fetal bovine serum (Thermo Fisher Scientific, Waltham, MA, USA), 100 U/mL penicillin and 100 mg/mL streptomycin at 37 °C with 5% of CO_2_. In addition, the ADSCs separated from rats differentiated into Schwann cells (SCs) as descripted in previous study [2]. The normal SCs acted as a control.

### Cell treatment and transfection

ADSCs (at 80% confluence) were treated with ICAII in different concentrations (10^−9^–10^−5^ mol/L), The cells without ICAII treatment was as a control. In addition, let-7i mimic (let-7i), negative control mimic (miR-NC), let-7i inhibitor (in-let-7i), negative control inhibitor (in-miR-NC), pcDNA3.0 vector (pcDNA), or STAT3 overexpression plasmid (STAT3) were also transfected into ADSCs with or without ICAII (10^−7^ mol/L) treatment. The treated or transfected cells were used for following experiments.

### Quantitative reverse transcription-polymerase chain reaction (qRT-PCR) assay

Total RNA was extracted from the cultured cells using Trizol reagent (Invitrogen, Carlsbad, CA, USA) according to the manufacturer’s instructions. For let-7i expression, total RNA was reversely transcribed into cDNA using One Step Prime Script miRNA cDNA Synthesis kit (Qiagen, Valencia, CA, USA). To quantify the level of STAT3, nerve growth factor (NGF), neurotrophin-3 (NT-3), S100β, and P75 neurotrophin receptor (P75), total RNA was reversely transcribed into cDNA using the prime-script reagent kit (Takara, Dalian, China). qRT-PCR was performed using SYBR green (Biosystems, Foster City, CA, USA). The primers were listed as follows: STAT3 forward, 5′-CACCCATAGTGAGCCCTTGGA-3′, and reverse, 5′-TGAGTGCAGTGACCAGGACAGA-3′; NGF forward, 5′-CCAAGGACGCAGCTTTCTATC-3′, and reverse, 5′-CTGTGTCAAGGGAATGCTGAAG-3′; NT-3 forward, 5′-TGTGGGTAGCCGACAAGTC-3′, and reverse, 5′-GAGTTCCAGTGTTTGTCATC-3′; S100β, forward 5′-TCACTGAGGGACGAAATCAACAC-3′, and reverse, 5′-GGTGCTATTGGTAGTCTGCCTTG-3′; P75 forward, 5′-GAGGGCACATACTCAGACGA-3′, and reverse, 5′-CTCTTCGCATTCAGCATCAG-3′; β-actin forward, 5′-ATGGATGACGATATCGCTGC-3′, and reverse, 5′- CTTCTGACCCATACCCACCA-3′; let-7i forward, 5′-GGGGTGAGGTAGTAGTTTGT-3′, and reverse, 5′-TGCGTGTCGTGGAGTC-3′; U6 forward, 5′-CTCGCTTCGGCAGCACA-3′, and reverse, 5′-AACGCTTCACGAATTTGCGT-3′. U6 and β-actin were used as internal controls for miRNAs or mRNAs, respectively. Relative expression was calculated using the 2^−∆∆Ct^ method.

### Western blot assay

Protein form treated or transfected cells were collected and subsequently measured using BCA Protein Assay Kit (Solarbio, Beijing, China). Then, each protein sample was separated by sodium dodecyl sulfate-polyacrylamide gel electrophoresis and then transferred onto polyvinylidene difluoride membranes (Roche Diagnostics, Indianapolis, IN, USA). After that, the membranes were blocked with 5% BSA for 1 h and then incubated with the primary antibody solution (STAT3, NGF, NT-3, S100β, P75, or β-actin; Abcam, Cambridge, MA, USA) at 4 °C overnight. The blots were probed by horseradish peroxidase-conjugated secondary antibody solution (Abcam) for 1 h. Finally, protein bands were visualized using PierceTM ECL western blotting substrate (Thermo Fisher Scientific). Captured the signals using films in dark room.

### 3-(4,5-Dimethyl-2-thiazolyl)-2,5-diphenyl-2-H-tetrazolium bromide (MTT) assay

Cell proliferation was measured by MTT assay. Briefly, the treated or transfected cells were seeded into 96-well plates and cultured for 48 h. MTT reagent solution (Thermo Fisher Scientific) was added to each well and incubated at 37 °C in 5% CO_2_ for 4 h. The optical density (OD) was detected at a wavelength of 450 nm using an Elx800 reader (Bio-Tek Instruments Inc., Winooski, VT, USA).

### Luciferase reporter assay

The 3′-UTR of STAT3 containing let-7i binding sites were amplified by PCR and inserted into pMIR-REPORT™ (Thermo Fisher Scientific) to construct STAT3 wild-type reporter vector (STAT3-WT). The mutant type (STAT3-MUT) was made with GeneArt™ Site-Directed Mutagenesis PLUS System (Thermo Fisher Scientific). STAT3-WT or STAT3-MUT and let-7i mimic, inhibitor or their negative controls were transfected into ADSCs using Lipofectamine 3000 (Thermo Fisher Scientific). The luciferase activity was examined using the Dual Luciferase Reporter Assay System (Promega, Madison, WI, USA) according the protocols. The transfections were performed three in independent experiments.

### Construction of the rat model with BCNI

Adult male SD rats (280–300 g, 9–10 weeks) were divided into six groups (n = 6): sham, BCNI, PBS, ADSCs, ICAII, and ADSCs + ICAII. The BCNI rat models were established as descripted in previous study [2]. Briefly, SD rats were anesthetized with pentobarbital sodium (Xinya Pharmaceutical Co., Ltd., Shanghai, China). In the sham group, the abdomen was then closed. In BCNI model group, the major pelvic ganglion and cavernous nerve were exposed on either side of the prostate, then direct perturbated the nerve 5 mm distal to the MPG using mosquito hemostatic forceps for 1 min. In PBS or ADSCs group: After the penis was exposed, an elastic band was applied to the base of the penis and maintained for 2 min, and a 1 × 106 rat ADSCs suspension in PBS (ADSCs group) or PBS alone (PBS group) was injected into both corpora cavernosa. In ICAII or ADSCs + ICAI group, ICAII (5 mg/kg/day) were treated with intragastric administration. The individual performing the BCNI blinded as to which rats would be receiving ICAII treatment.

### Erectile function evaluation

Mean arterial pressure (MAP) and intracavernosal pressure (ICP) were applied to evaluate the EF of the rats at 4 weeks after the surgery and injection as descripted in previous study [2]. EF was analyzed with the ratio of ICP/MAP. The stimulation parameters were 16 Hz with duration of 5 ms at 5 V for 60 s with 5 min between subsequent stimulations.

### Statistical analysis

Data were presented as the mean ± standard deviation. The significance of the in vitro data and in vivo data between experimental groups was determined by Student’s *t* test or one-way ANOVA. *P *< 0.05 was considered to be statistically significant.

## Results

### ICAII promotes the proliferation and differentiation of ADSCs

**Fig. 1:**
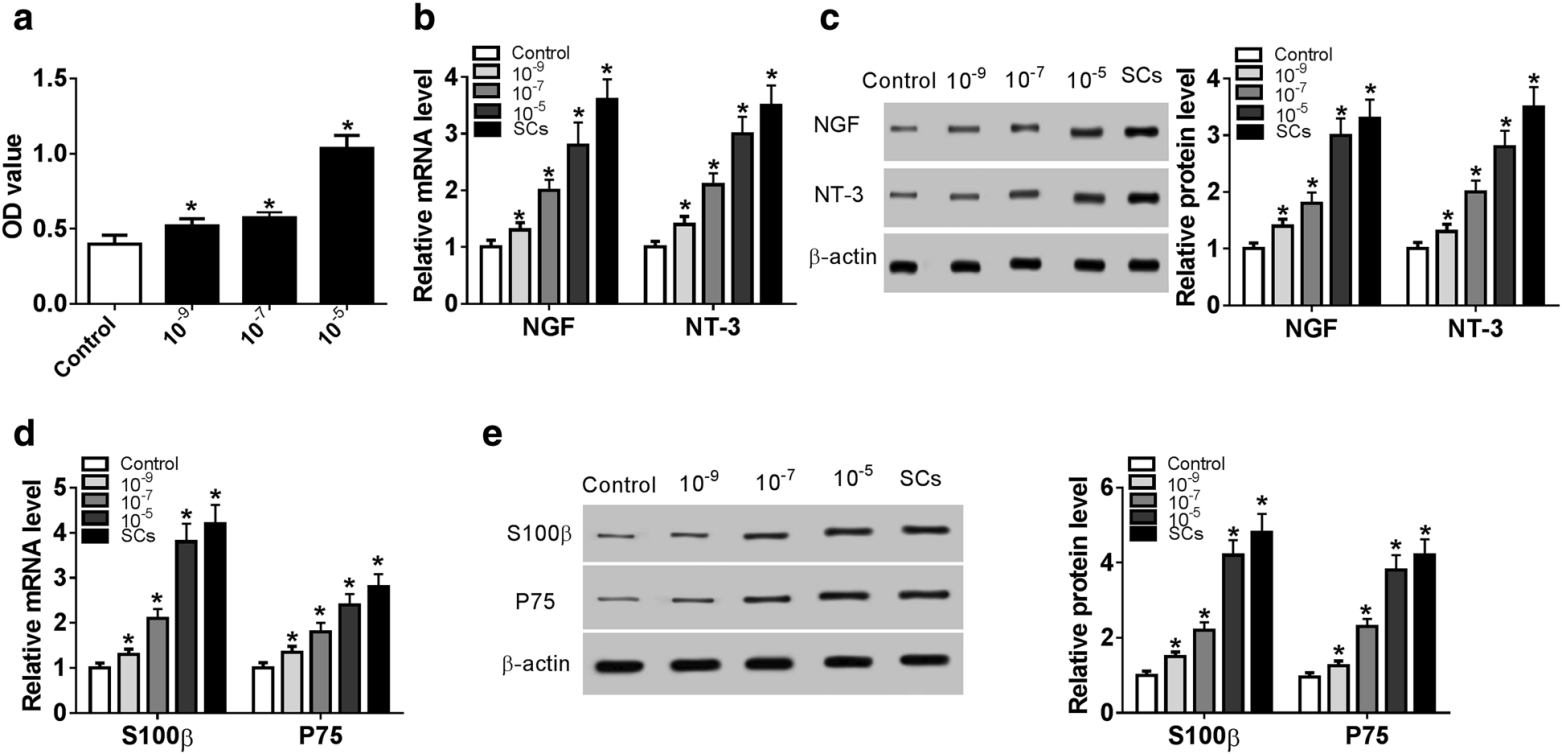
ICAII promotes the proliferation and differentiation of ADSCs. ADSCs were treated with various concentration of ICAII (10^−9^, 10^−7^, and 10^−5^ mol/L), and the normal SCs separated from the rats served as a control. **a** Cell proliferation was determined by MTT assay. **b**, **c** qRT-PCR and western blot were performed to detect the expression levels of NGF and NT-3. **d**, **e** qRT-PCR and western blot were conducted to measure the expression levels of S100β and P75. *P < 0.05

Firstly, ADSCs were isolated from adipose tissues of SD rats. We found that cell proliferation of ADSCs was promoted by ICAII in a dose-dependent manner (Fig. 1a). To further investigate the effect of ICAII on differentiation of ADSCs to SCs, the expressions of SCs markers such as NGF, NT-3, S100β, and P75 were subsequently examined. ICAII increased the mRNA and protein levels of NGF and NT-3 in a dose-dependent manner (Fig. 1b, c). Similarly, the ICAII treatment group had a higher amount of S100β, and P75 than that of control group (Fig. 1d, e). Those data suggested that ICAII could promote the proliferation and differentiation of ADSCs.

### ICAII stimulates the differentiation of ADSCs to SCs through inhibiting let-7i

**Fig. 2:**
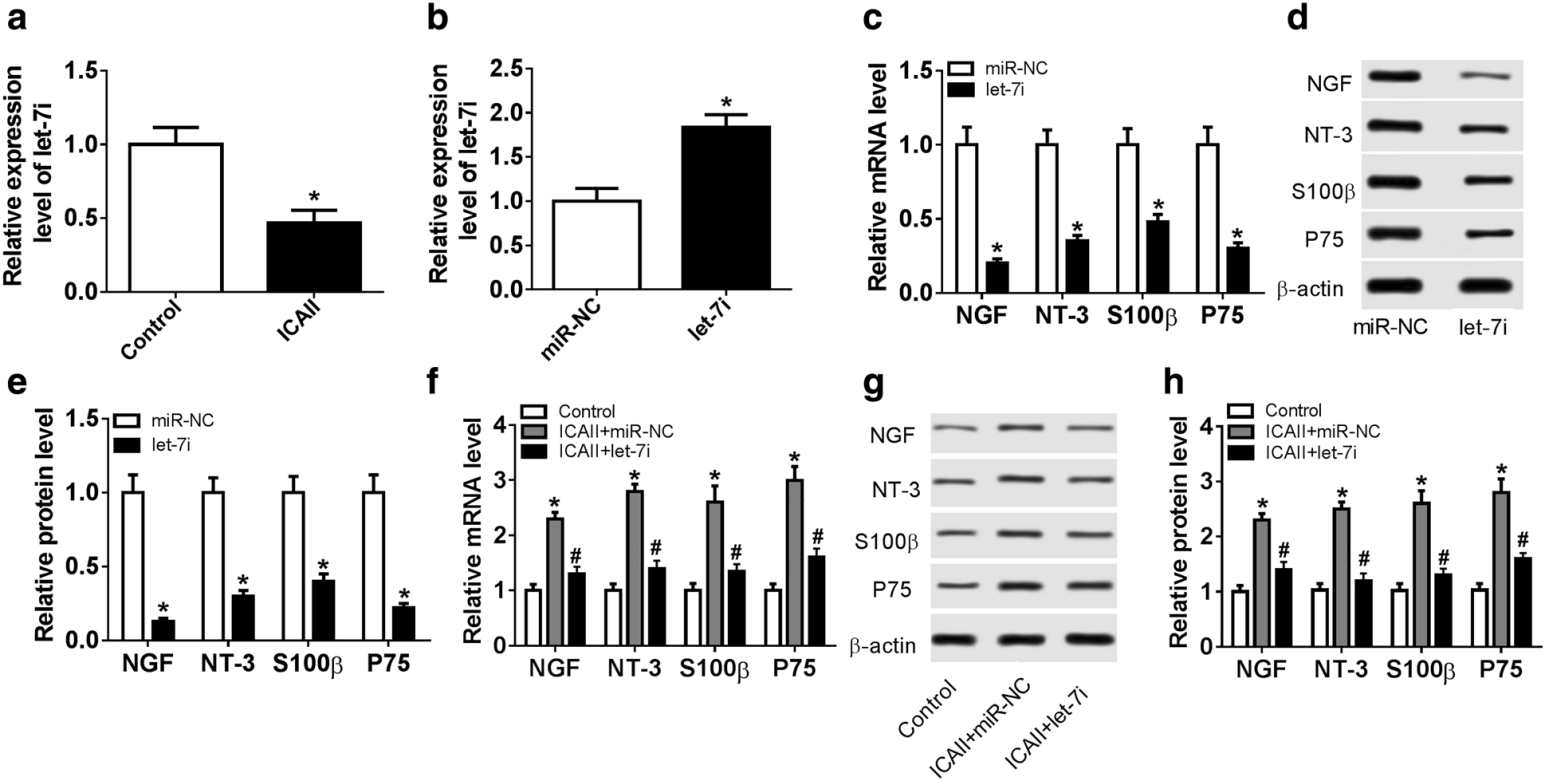
ICAII stimulates the differentiation of ADSCs to SCs through inhibiting let-7i. **a** qRT-PCR was performed to measure the level of let-7i in ADSCs treated with ICAII (10^−7^ mol/L). **b** ADSCs were transfected with let-7i mimic or negative control mimic and the level of let-7i was determined by qRT-PCR. **c** qRT-PCR was conducted to measure the mRNA levels of NGF, NT-3, S100β, and P75. **d**, **e** Western bolt was performed to measure the protein levels of NGF, NT-3, S100β, and P75. **f**–**h** let-7i mimic and negative control mimic were transfected into ADSCs treated with ICAII (10^−7^ mol/L). Western bolt and qRT-PCR were conducted to measure the expression levels of NGF, NT-3, S100β, and P75. *P < 0.05, ^#^*P *< 0.05

Then, we explored whether let-7i is implicated in the ICAII-mediated SCs differentiation of ADSCs. As presented in Fig. 2a, let-7i was decreased in ADSCs after ICAII treatment, revealing that the inhibition of let-7i may participate in the process of differentiation of ADSCs to SCs. Therefore, the role of let-7i in this process was further addressed. The qRT-PCR analysis demonstrated that the level of let-7i was higher in cells transfected with let-7i mimic than that in miR-NC group (Fig. 2b). Moreover, upregulation of let-7i reduced the mRNA and protein levels of SCs markers (Fig. 2c–e). Besides, the effect of let-7i on the ICAII-induced differentiation of ADSCs to SCs was further validated. The results showed that the introduction of let-7i mimic attenuated the ICAII-induced differentiation of ADSCs to SCs (Fig. 2f–h), indicating that ICAII may stimulate the differentiation of ADSCs to SCs via inhibiting let-7i.

### STAT3 is a target gene of let-7i

**Fig. 3:**
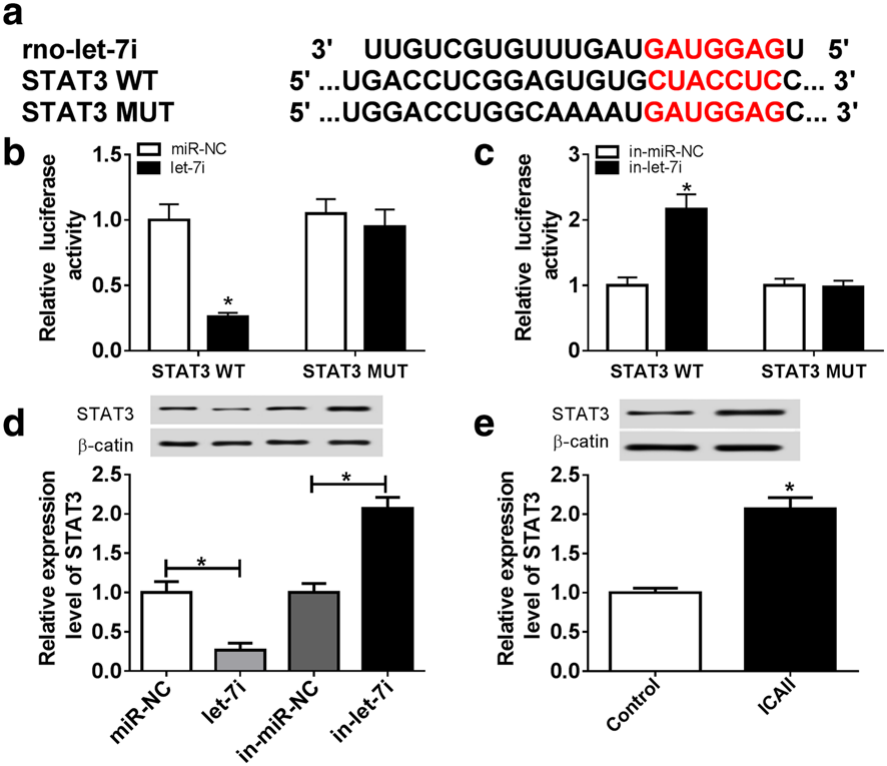
STAT3 is a target gene of let-7i. **a** The binding sites between let-7i and STAT3 were predicted by Targetscan online database and the luciferase reporter plasmids containing the wild-type (WT) or mutated (MUT) STAT3 binding sites of let-7i were established. **b** The luciferase activity was measured in ADSCs co-transfected with STAT3-WT or STAT3-MUT luciferase reporter and let-7i mimic or negative control mimic **c** The luciferase activity was measured in ADSCs co-transfected with STAT3-WT or STAT3-MUT luciferase reporter and let-7i inhibitor or negative control inhibitor. **d** Western blot was conducted to measure protein level of STAT3 in ADSCs transfected with let-7i mimic, inhibitor, or their negative controls. **e** Western blot was performed to measure the protein level of STAT3 in ICAII (10^−7^ mol/L)-treated ADSCs. *P < 0.05

We next dissected the potential molecular mechanism by which let-7i repressed the differentiation of ADSCs to SCs. miRNAs regulate gene expression by posttranscriptional in a sequence-specific manner [21]. Targetscan online database predicted that the 3′UTR regions of STAT3 contains potential let-7i binding sites (Fig. 3a). In view of this, we hypothesized that STAT3 may be a target of let-7i. Then, luciferase reporter assay was introduced to identify our hypothesis. As expected, a significant repression of luciferase activity was observed upon transfection with let-7i mimic in STAT3 WT group (Fig. 3b). In contrast, let-7i depletion increased the luciferase activity in STAT3 WT group, while no significant change was observed in STAT3 MUT group (Fig. 3c). Overexpression of let-7i resulted in the decrease expressions of STAT3. However, the silencing of let-7i led to an opposite effect (Fig. 3d). These data lend credence to our hypothesis that let-7i directly bind to STAT3. In the following experiments, we determined the effect of ICAII on the expression of STAT3. The results of western blot declared that STAT3 expression was upregulated in cells with ICAII treatment (Fig. 3e).

### STAT3 is involved in ICAII/let-7i-mediated differentiation of ADSCs to SCs

**Fig. 4:**
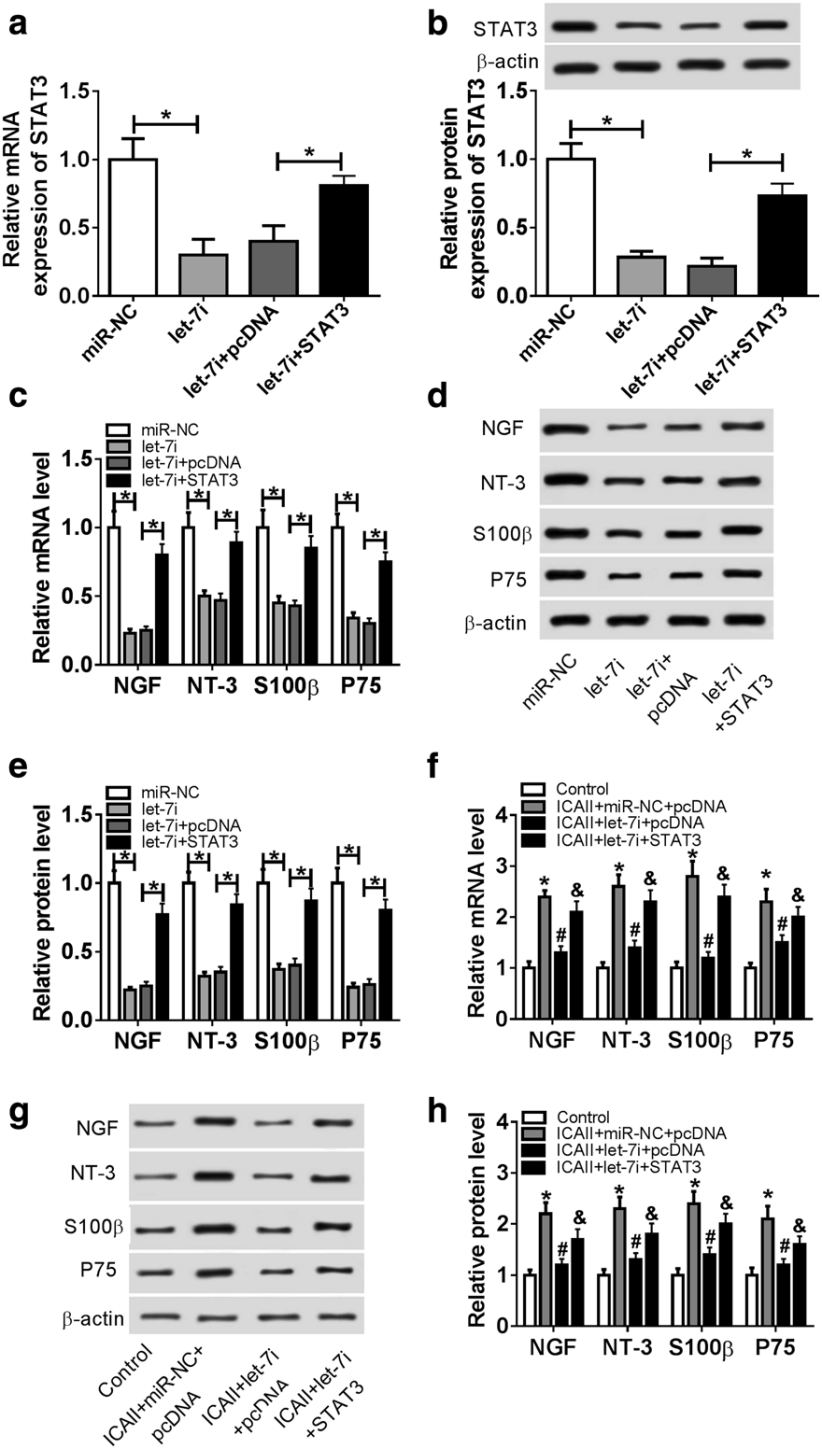
STAT3 is involved in ICAII/let-7i-mediated differentiation of ADSCs to SCs. **a**, **b** ADSCs were transfected with negative control mimic, let-7i mimic, let-7i mimic + pcDNA 3.0 vector, or let-7i mimic + STAT3 overexpression plasmid. qRT-PCR and western blot were performed to measure the expression level of STAT3 in ADSCs. **c**–**e** The expression levels of NGF, NT-3, S100, and P75 in ADSCs were determined by qRT-PCR and western blot. **f** Negative control mimic + pcDNA 3.0 vector, let-7i mimic + pcDNA 3.0 vector, or let-7i mimic + STAT3 overexpression plasmid were transfected into ADSCs after ICAII (10^−7^ mol/L) treatment. qRT-PCR was employed to detect the mRNA levels of NGF, NT-3, S100, and P75 in ADSCs. **g**, **h** The protein levels of NGF, NT-3, S100, and P75 in ADSCs were determined by western blot. *P < 0.05, ^#^*P *< 0.05, ^&^*P *< 0.05

Given the above results, we next sought to evaluate the role of STAT3 in the differentiation of ADSCs to SCs. Also, following transfection efficiency demonstrated that the transfection of let-7i mimic was efficient to induce the lower expression of STAT3, while the introduction of STAT3 overexpression plasmid strikingly rescued STAT3 expression in ADSCs (Fig. 4a, b). Moreover, let-7i upregulation suppressed the expressions of SCs markers, which were rescued by enforced expression of STAT3 (Fig. 4c–e), referring that let-7i may be response for differentiation of ADSCs to SCs by downregulating STAT3. In addition, we further unveiled that overexpression of STAT3 abrogated the inhibition effect of let-7i on ICAII-mediated promotion of SCs markers, thus leading to the differentiation of ADSCs to SCs (Fig. 4f–h).

### Combination treatment of ADSCs and ICAII preserves the EF of the BCNI model rats

**Fig. 5:**
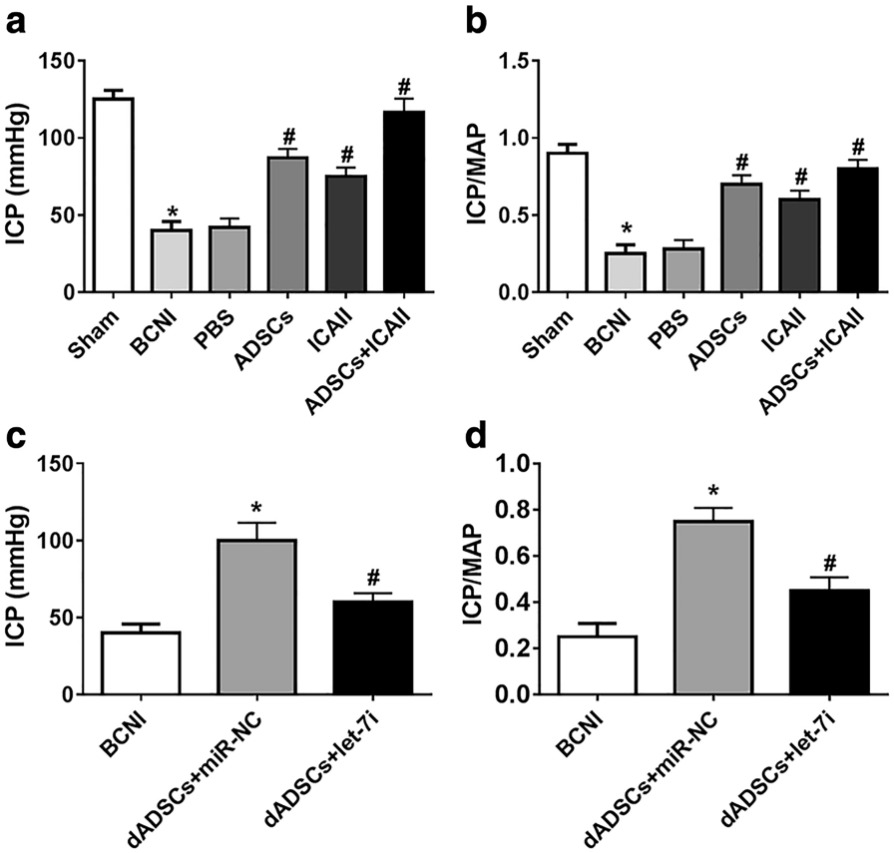
Combination treatment of ADSCs and ICAII preserves the EF of the BCNI model rats. **a**, **b** SD male rats were divided into six groups: Sham, BCNI, PBS, ADSCs, ICAII, and ADSCs + ICAII. The detection of ICP and MAP and the ratio of ICP/MAP were conducted to evaluate the EF of the rat models. **c**, **d** SD male rats were divided into three groups: BCNI, dADSCs (ADSCs were treated with ICAII) +negative control mimic, or dADSCs + let-7i mimic. The detection of ICP and MAP and the ratio of ICP/MAP were conducted to evaluate the EF of the rat models. *P < 0.05, ^#^*P *< 0.05

Finally, BCNI rat models were established to identify the roles of ICAII and ADSCs. As shown in Fig. 5a, b, ICP and the ratio of ICP/MAP were distinctly lower in BCNI group than that of sham group, whereas both of them were increased after ADSCs or ICAII treatment. More importantly, those effects were enhanced by combination of ICAII and ADSCs. Intriguingly, upregulation of let-7i curbed dADSCs-induced promotion of ICP value and the ratio of ICP/MAP (Fig. 5c, d). Taken together, our data indicated that the combination of ICAII and ADSCs preserved the EF of the BCNI model rats.

## Discussion

ICAII, an active component of Herba Epimedii, has been proven to improve cardiovascular function and facilitate the differentiation of ADSCs to cardiomyocytes [22, 23]. In this report, our results showed that ICAII stimulated ADSCs proliferation. ADSCs are pluripotent stem cells isolated from the adipose tissue and have the potential for self-renewal and multi-directional differentiation [24, 25]. Moreover, it has been reported that ADSCs can promote EF and cavernous structures in ED rats [26]. A study also indicated that ADSCs could differentiate into SCs and the harvested SCs could be used for cell-based treatment of various peripheral nerve injuries or disorders [27, 28]. Furthermore, SCs also play vital roles in the regeneration of penile and peripheral nerves [29]. NGF, NT-3, S100β, and P75 are widely used as markers of SCs [2, 30]. In the present study, we further demonstrated that ICAII enhanced the expressions of NGF and NT-3, S100β, and P75 in ADSCs. Those data suggested that ICAII could promote the proliferation and differentiation of ADSCs to SCs.

miRNAs play critical roles in regulating the self-renewal and differentiation of ADSCs [31]. Previous study has proved that miRNAs could regulate the myelin formation of SCs response to nerve damage [32]. Yi et al. have reported that miR-30c promotes SCs remyelination after peripheral nerve injury [33]. Interestingly, we found that the level of let-7i was downregulated in ADSCs following ICAII treatment. Hence, we thought that let-7i may have an implication in the ICAII-mediated function in ADSCs. Moreover, whether let-7i is involved in ICAII-mediated differentiation of ADSCs was further investigated. We disclosed that overexpression of let-7i weakened the ICAII-mediated promotion effect on differentiation of ADSCs to SCs. Those data implied that ICAII may promote the differentiation of ADSCs to SCs through inhibiting let-7i.

Proteins of the signal transducer and activator of transcription (STAT) family mediate cellular responses to cytokines and growth factors [34]. STAT3 is crucial for progression of cancer and promotes cancer stem cells self-renewal and differentiation [35]. Moreover, STAT3 was reported to be a contributor to maintain the molecular and morphological repair phenotype that promotes axonal regeneration of SCs [36]. In another study, it has been revealed that STAT3 is required for promoting synthetic phenotype transition and contributes to ED therapy [19]. Furthermore, recent study also reported that ADSCs are promoted by STAT3 to differentiate into SCs [2]. In the current report, we found that let-7i negatively regulated the expression of STAT3. Importantly, STAT3 was upregulated in ADSCs following ICAII treatment, which was consistent with previous study [10]. Intriguingly, the results further disclosed that overexpression of STAT3 counteracted the block effect of let-7i on ICAII-mediated promotion of differentiation of ADSCs to SCs. Those findings indicated that let-7i/STAT3 axis was responsible for the ICAII-induced the differentiation of ADSCs to SCs in vitro. In addition, the results of in vivo further fueled the conclusion that upregulation of let-7i mitigated the effect of dADSCs on EF, which was in line with the results of vitro. More importantly, the combination of ICAII and ADSCs on effect of the preserving EF was also verified in BCNI rat models. The data suggested that combination treatment of ADSCs and ICAII preserves the EF of the BCNI model rats.

In conclusion, our results demonstrate for the first time that ICAII facilitates the differentiation of ADSCs to SCs via let-7i/STAT3 axis to preserve erectile function. However, there are some limitations in this report. Our study only shed light on the roles of let-7i and STAT3 in the treatment of ICAII and ADSCs for ED, further research is needed to study the underlying signal pathways.

## Conclusion

In summary, we found that ICAII promoted differentiation of ADSCs to SCs and ICAII was responsible for preserving EF after CNI by promoting the differentiation of ADSCs to SCs via modulating let-7i/STAT3 axis. Moreover, let-7i suppressed the therapeutic effect of dADSCs on preserving EF in BCNI rat models. Our findings provided novel insights into the let-7i/STAT3 axis in ICAII-mediated promotion of differentiation of ADSCs to SCs and a significant theoretical basis for the utilization of ICAII for ED treatment.

## Data Availability

All data generated and analyzed during this study are included and available.

## Abbreviations

ICAII: IcarisideII
SCs: Schwann cells
EF: erectile function
ICP: intracavernosal pressure
BCNI: bilateral cavernous nerve injury
ED: erectile dysfunction
ICAII: Icariside II
MAP: mean arterial pressure
ICP: intracavernosal pressure
